# Intravenous lidocaine for post-operative pain relief after hand-assisted laparoscopic colon surgery: a randomized, placebo-controlled clinical trial

**DOI:** 10.1007/s10151-013-1065-0

**Published:** 2013-09-13

**Authors:** R. Tikuišis, P. Miliauskas, N. E. Samalavičius, A. Žurauskas, R. Samalavičius, V. Zabulis

**Affiliations:** 1 Institute of Oncology, Vilnius University, Santariškių Str. 1, 08660 Vilnius, Lithuania; 2 Faculty of Medicine, Clinics of Anesthesiology and Intensive Care, Vilnius University, Šiltnamių Str. 29, 04130 Vilnius, Lithuania; 3 Faculty of Medicine, Clinics of Internal Diseases, Family Medicine and Oncology, Vilnius Uuniversity, Santariškių Str. 2, 08661 Vilnius, Lithuania; 4 II Department of Anesthesia, Intensive Care and Pain Management, Vilnius University Hospital Santariškių Clinics, Santariškių Str. 2, 08660 Vilnius, Lithuania; 5 Department of Cardiovascular Disease, Vilnius University, Santariškių Str. 2, 08661 Vilnius, Lithuania; 6 Center of Reconstructive and Endovascular Surgery, Vilnius University Hospital Santariškių Clinics, Santariškių Str. 2, 08661 Vilnius, Lithuania

**Keywords:** Acute pain, Analgesics, Post-operative, Lidocaine, Laparoscopic, Hemicolectomy, Hand-assisted laparoscopic colonic surgery (HALS)

## Abstract

**Background:**

Perioperative intravenous (IV) infusion of lidocaine has been shown to decrease post-operative pain, shorten time to return of bowel function, and reduce the length of hospital stay. This randomized, prospective, double-blinded, placebo-controlled clinical trial evaluated the impact of IV lidocaine on the quality of post-operative analgesia and other outcomes after hand-assisted laparoscopic colon surgery.

**Methods:**

Sixty four patients with colon cancer scheduled for elective colon resection were involved in this study. Patients were randomized to receive either lidocaine infusion [lidocaine group (LG)] or normal 0.9 % saline infusion [placebo group (PG)] for a period of 24 h. Anaesthetic and surgical techniques were standardized. Twenty-four-hour post-operative analgesia in the recovery area was maintained by continuous infusion of 0.1 μg/kg/h fentanyl. The primary outcome of the study was post-operative pain control. Pain was assessed using visual analogue scale (VAS) scores at 2, 4, 8, 12, and 24 h after surgery. Patients with a VAS score >3 were treated with ketorolac 30 mg as needed. Secondary outcomes included time to resumption of bowel function and length of hospital stay. Data in the two groups were compared using the two-tailed Student’s *t* test. All statistical tests were two-tailed at a significance level of 0.05.

**Results:**

Demographic characteristics and clinical features of both groups were similar. Intensity of pain at rest in LG compared with PG was significantly lower during the first 24 h post-operatively. LG patients reported significantly less pain during movements at 2-, 12-, and 24-h post-surgery than PG patients. The study showed that ketorolac consumption was significantly higher in PG: mean ketorolac consumption in LG was 43.77 ± 13.86 mg and in PG 51.67 ± 13.16 mg (*p* = 0.047). Compared with placebo, lidocaine infusion produced a 32 % reduction in time to the first drink (Cohen’s *d* = 3.85), 16 % reduction in time to the first full diet (Cohen’s *d* = 3.35), and 18 % reduction in time to the first bowel movement (Cohen’s *d* = 2.30). Patients who received lidocaine stayed in hospital 1.2 days less than patients who received placebo (*p* < 0.01, Cohen’s *d* = 0.72). There were no significant differences in surgery-related complications between the two groups.

**Conclusions:**

Perioperative continuous IV lidocaine infusion has a beneficial effect as regards post-operative pain, restoration of bowel function, and length of hospital stay in patients who have undergone hand-assisted laparoscopic colon surgery.

## Introduction

Clinical and scientific data show that laparoscopic methods in colon surgery accelerate dietary intake and return of bowel function, facilitate post-operative mobilization, reduce the length of stay in hospital, and reduce the post-operative mortality rate [1–6]. A number of meta-analyses and systematic reviews proved the safety of hand-assisted laparoscopic surgery (HALS) for patients with colon malignancies [1–6].

Furthermore, post-operative pain management in the treatment of acute pain after HALS (compared with conventional surgery) improves both the success of the surgical intervention and patient comfort [5–7]. To date no standard method of post-operative analgesia for patients undergoing HALS has been developed, and this has led to the employment of various types of analgesia. These have included epidural analgesia, patient-controlled analgesia, intravenous (IV) agents, and spinal anaesthesia [8].

Recently, there has been a surge of interest in the use of IV lidocaine in abdominal surgery due to its analgesic, antihyperalgesic, and anti-inflammatory effects. It has been shown that perioperative administration of IV lidocaine decreases post-operative pain, minimizes the use of opioids, facilitates earlier restoration of bowel function, and shortens the length of hospital stay after colon surgery [9, 10].

The aim of this study was to assess the effectiveness of IV lidocaine versus placebo on the post-operative pain level, requirements for analgesics, duration of post-operative ileus, and length of hospital stay in patients undergoing hand-assisted laparoscopic colon surgery.

## Materials and methods

This randomized prospective double-blinded placebo-controlled single-centre trial was performed at the Institute of Oncology, Vilnius University, Lithuania. The study was approved by the Local Ethics Committee and written informed consent was obtained from all patients.

### Study population

Consecutive adult patients 18–75 years old with colon cancer scheduled for an elective laparoscopic colon resection under general anaesthesia were invited to participate in this study. Patients were enroled from March 2010 until March 2012. Patients American Society of Anaesthesiologists (ASA) score of I, III, or III, with normal cognitive function and able to give informed consent, were eligible for inclusion in the study. Patients with severe hepatic, renal, cardiac, respiratory, and endocrine disease and history of alcohol or drug addiction, those taking analgesics pre-operatively and those with allergy to local anaesthetic were excluded from the study.

The study hypothesis was that lidocaine infusion would result in better pain relief, earlier resumption of bowel function, and a shorter hospital stay in patients undergoing hand-assisted laparoscopic colon surgery.

### Anaesthesia technique and lidocaine administration

For all patients, anaesthetic management was standardized and based on the standards of care adopted in our institution. General anaesthesia was provided to all patients using fentanyl 1.5 μg/kg and propofol 2 mg/kg for induction, and tracheal intubation was achieved with rocuronium. Intraoperative muscle relaxation was monitored using a nerve stimulator. Arterial blood pressure and heart rate were maintained within 20 % of baseline values. Supplemental doses of 50 μg of fentanyl were administered if intraoperative blood pressure or heart rate were 20 % higher than baseline. Intraoperative blood pressure or heart rate 20 % lower than baseline was treated with IV ephedrine. Anaesthesia was maintained with sevoflurane in a mixture of air 40 % and oxygen 60 %, and end-tidal concentration of sevoflurane was adjusted to maintain the bispectral index within range, between 40 and 60. Twenty-four-hour post-operative analgesia in the recovery area was maintained by continuous infusion of 0.1 μg/kg/h fentanyl.

For LG patients, an IV bolus of lidocaine 1.5 mg/kg was given (maximum 100 mg) just before the induction of anaesthesia, followed by an IV infusion of lidocaine 2 mg/kg/h during the entire surgical procedure. The dose of lidocaine was then lowered to 1 mg/kg/h in the post-operative anaesthesia care unit and continued for the first 24 h after surgery. PG patients received the same amount of pre-operative bolus and continuous infusion of normal saline during surgery and for 24 h after the operation.

### Surgical technique

All hand-assisted laparoscopic colon operations were carried out by the same team of surgeons experienced in this procedure. With the patient under general anaesthesia, in a supine horizontal position with legs stretched, body fixed to the operating table and operator standing between the stretched legs, a 6–6.5 cm transumbilical incision is performed. The assistant is standing on the right side of the patient and the scrub nurse on the left side. The laparoscopic port is inserted and a left hand is introduced into the abdomen. Under hand control, a 5-mm trocar is inserted in the left lateral quadrant few centimetres above and towards the midline from the anterior superior iliac spine, and a 10-mm trocar is inserted at the level of the right midcalvicular line few centimetres above the umbilicus (camera port, to allow visualization of both the splenic flexure and transverse colon, and the pelvic area). A 12-mm trocar is inserted 2–3 cm towards the midline and 2–3 cm below the right anterior superior iliac spine. Mobilization begins with the descending colon moving upwards to splenic flexure and left side of transverse colon, using a hand and a harmonic scalpel (cranial part elevated and patient turned to the right). After this part is finalized, the operator moves to the right side of the patient (same as assistant surgeon) and mobilization continues with the sigmoid colon, then lifting the rectosigmoid colon at the level of the promontorium with superior rectal vessels and continuing the mobilization from the left side, using a 12-mm trocar for the harmonic scalpel, visualizing the left ureter (caudal part elevated and turned to the right). Then, the inferior mesenteric artery is mobilized and ligated using titanium 10-mm clips 1–2 cm from the aorta, and the inferior mesenteric vein is mobilized and ligated with same clips at the level of the ligament of Treitz. The specimen is divided at the level of the promontorium, using an endoscopic linear stapler (60 mm). The specimen is removed through the hand port incision, and further anastomosis is fashioned laparoscopically using a double-stapling technique. The water–air leak test is performed and the doughnuts are examined to ensure completeness. No drain is routinely used. The fascia is closed at the level of the 12-mm trocar with a single interrupted suture, and the hand port with a running polydioxanone 0 suture. The skin incisions are closed with interrupted sutures.

### Randomization and blinding

Patients were allocated to LG or PG before surgery using a computer-generated randomization list of random numbers. An anaesthesia nurse not involved in the patients’ treatment and evaluation drew lidocaine or normal saline into two syringes labelled with each patient’s number and the administration route: for bolus and for infusion. Patients and those who gathered data (treating surgeons, anaesthesiologist, and nurse) were blinded to study allocation.

### Outcome measures

The primary outcomes of the study were post-operative pain control evaluated by determining the intensity of pain and ketorolac consumption. The intensity of abdominal pain at rest and during movement (i.e. deep breathing, coughing) was assessed regularly at 2, 4, 8, 12, and 24 h after the operation using visual analogue scale (VAS) pain scores (from 0 to 10: 0 means no pain, whereas a score of 10 equals the worst pain ever experienced). Patients received an information sheet and verbal training on completing the pain scores. Patients with a VAS score >3 were treated with IV ketorolac 30 mg as needed. Ketorolac consumption was registered.

Secondary outcomes included time to resumption of bowel function and length of hospital stay. All patients were instructed to report the time of the first flatus and bowel movement. The patient informed the nursing staff, who documented the time. Patients were discharged from the hospital after pain control using oral pain medication had been optimized, bowel function had resumed, and they had started to tolerate a solid diet, had become mobile, and were able to perform daily activities independently or with minimal aid. There were no changes in trial outcomes after the start of the study.

Data collected as well included surgery-related complications (ileus, nausea and vomiting, urinary retention requiring insertion of a urinary catheter, anastomotic leak, and wound infection).

### Statistical analysis

The primary outcome variable was the VAS pain score after surgery. A previous study had been conducted where VAS pain scores at rest and at movements were recorded 2, 4, 8, 12, and 24 h after hand-assisted laparoscopic colon surgery for 15 patients who had received IV normal saline [11]. The biggest standard deviation (SD) of the scores in this group was 1.6. A similar SD was assumed for patients receiving IV lidocaine. To estimate the group size needed to show statistical significance, assuming a between-group difference in VAS pain score of 1.3 as reported by Gallagher et al. [12] with a two-tailed *α* = 0.05 and power of 80 %, it was calculated that a minimum of 24 patients/group was required. One of the secondary outcomes was time to the first bowel movement. The average time to return of bowel movements in the lidocaine subgroup of the study of Groudine et al. [13] was 61.8 ± 13.2 h. Sample size calculation confirmed that 24 patients in each group were sufficient to demonstrate a difference in time to return of the bowel movement with a two-tailed *α* = 0.05 and a power of 80 % [13]. The total number of patients was increased to 64 to include dropouts.

Continuous data [age, weight, height, body mass index (BMI), duration of surgery, duration of anaesthesia, pain scores, ketorolac consumption, time to the first drink, time to a full diet, time to the first bowel movement, and length of hospital stay] are presented as mean ± SD and 95 % confidence interval (CI) and analyzed using Student’s *t* test or the Mann–Whitney *U* test as appropriate. Data regarding patient gender, ASA score, and number of patients with surgery-related complications are presented as frequency and percentage and analyzed with the χ^2^ test or Fisher’s exact test as appropriate. To evaluate the relative impact of lidocaine infusion on pain control, ketorolac consumption, and time to resumption of bowel function, we estimated the effect size of each intervention through Cohen’s *d* test. Ketorolac consumption effect on pain scores and time to bowel functions restoration was assessed using a general linear model.

All statistical tests were two-tailed at a significance level of 0.05. Statistical analysis of data was performed using SPSS version 18 for Windows (SPSS Inc., Chicago, IL, USA).

## Results

**Fig. 1:**
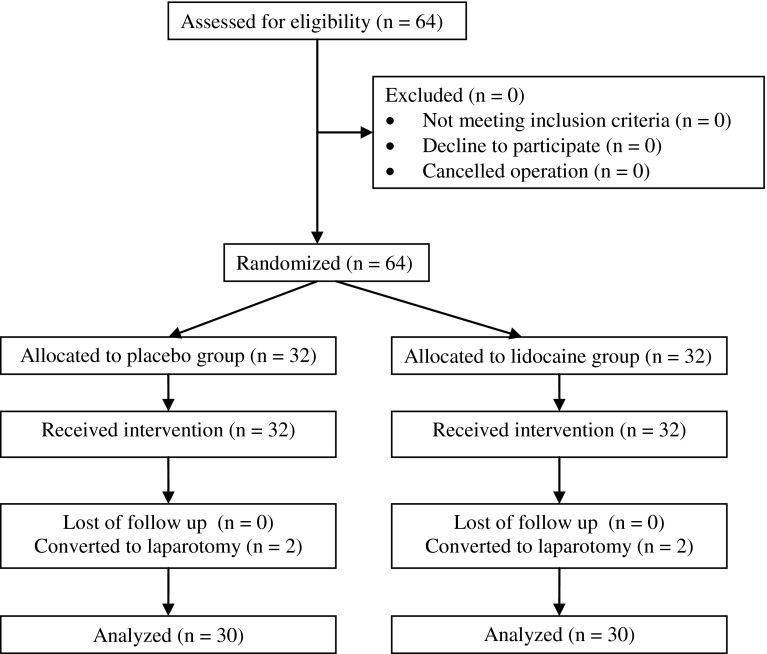
CONSORT diagram showing progress of participants through the study

Sixty-four consecutive adult patients (61.7 % male and 38.2 % female) with a diagnosis of colon cancer were recruited for the study. All patients underwent hand-assisted laparoscopic hemicolectomy. Thirty-two patients were randomized to receive lidocaine and 32 patients to receive normal saline as placebo. Four patients had to be excluded from final analysis because HALS was converted to laparotomy. The data of 60 patients were analyzed (Fig. 1). Mean age was 56.6 ± 12.7 years, mean BMI 24.5 ± 2.4 kg/m^2^.

The lidocaine and placebo groups did not differ significantly with respect to age, gender, ASA score, height, weight, BMI, duration of anaesthesia, or duration of surgery (Table 1).

**Table 1:** Demographic characteristics and clinical data for lidocaine group and placebo group (mean ± SD or number of patients)

Variables	Lidocaine group (*n* = 30)	Placebo group (*n* = 30)	*p* value
Age (years)	57.20 ± 13.28	56.00 ± 12.22	0.717
Gender (male/female)	18/12	19/11	1.00
ASA score (I/II/III)	19/7/4	21/5/4	0.805
Height (cm)	174.97 ± 4.63	173.90 ± 4.96	0.393
Weight (kg)	73.00 ± 6.09	75.53 ± 5.69	0.101
BMI (kg/m^2^)	23.92 ± 2.62	25.01 ± 2.10	0.080
Duration of anaesthesia (min)	115.00 ± 10.91	114.33 ± 10.96	0.814
Duration of surgery (min)	113.67 ± 11.74	111.50 ± 10.68	0.458

*ASA* American Society of Anaesthesiologists, *BMI* body mass index, *SD* standard deviation

**Fig. 2:**
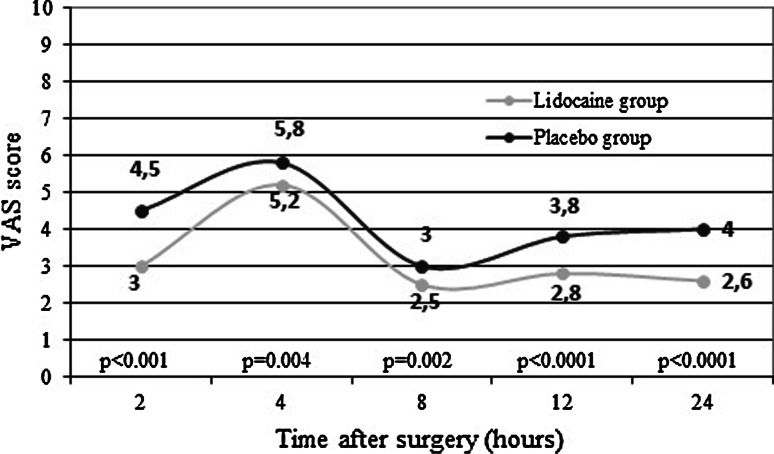
Comparison of mean visual analogue scale (VAS) scores in lidocaine and placebo groups at rest

Intensity of pain at rest in LG compared with PG was significantly lower during the first post-operative day at each evaluation (*p* < 0.01) (Fig. 2). Differences remained significant after adjusting for ketorolac usage.

**Fig. 3:**
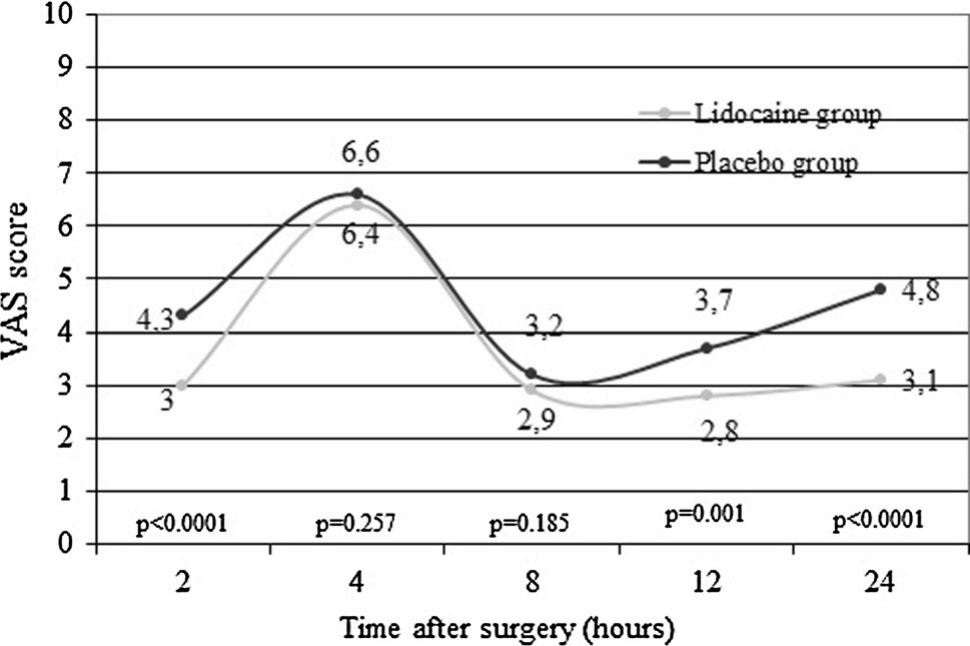
Comparison of mean visual analogue scale (VAS) scores in lidocaine and placebo groups during movement

LG patients reported significantly less pain during movements at 2-, 12-, and 24-h post-surgery than PG patients (*p* < 0.01) (Fig. 3). Difference remained significant after adjusting for ketorolac usage. Lidocaine infusion produced large effect on post-operative pain at rest and during movements.

Twenty-seven (90.0 %) of PG patients required ketorolac and 22 (73.3 %) of LG patients required ketorolac (*p* = 0.181). Patients receiving lidocaine consumed significantly less ketorolac: mean ketorolac consumed by patients receiving lidocaine was 43.77 ± 13.86 mg and PG patients received on average 51.67 ± 13.16 mg of ketorolac (*p* = 0.047, Cohen’s *d* = 0.58).

Clinical data from the post-operative period are presented in Table 2. Mean time to the first drink, to the first full diet, and to the first bowel movement was significantly shorter in LG than in PG (10 and 6 h respectively, *p* < 0.01). Differences in time remained significant after adjusting for ketorolac usage. Compared with placebo, lidocaine infusion produced a 32 % reduction in time to the first drink (Cohen’s *d* = 3.85), 16 % reduction in time to the first full diet (Cohen’s *d* = 3.35), and 18 % reduction in time to the first bowel movement (Cohen’s *d* = 2.30).

**Table 2:** Comparison of time to first drink, time to first full diet, first bowel movement, and length of hospital stay in lidocaine and placebo groups (mean ± SD)

Variables	Lidocaine group (*n* = 30)	Placebo group (*n* = 30)	Mean difference (95 % CI)	*p* value
Time to first drink (h)	21.03 ± 2.41	31.03 ± 2.77	−10.00 (−11.34 to −8.66)	<0.0001
Time to first full diet (h)	30.97 ± 1.83	36.97 ± 1.75	−6.00 (−6.93 to −5.08)	<0.0001
First bowel movement (h)	26.97 ± 2.30	32.93 ± 2.86	−5.97 (−7.31 to −4.63)	<0.0001
Length of hospital stay (days)	4.70 ± 1.29	5.90 ± 1.97	−1.2 (−2.06 to −0.34)	0.007

*SD* standard deviation

Patients who received lidocaine stayed in hospital 1.2 days less than patients who received placebo (*p* < 0.01, Cohen’s *d* = 0.72).

There were no complications directly related to anaesthesia observed in either group. Lidocaine-associated hemodynamic changes such as severe hypotension, bradycardia, and arrhythmia were not observed in any LG patient during surgery. In addition, during the post-anaesthesia pot care unit stay, no patient complained of lidocaine-induced toxicity such as light headedness, perioral numbness, metallic taste, dizziness, and visual disturbances. There were no significant differences in surgery-related complications (ileus or absence of bowel movements, nausea, vomiting, urinary retention, anastomotic leak, and wound infection) between the groups (Table 3).

**Table 3:** Incidence of surgery-related complications in lidocaine and placebo groups (number of patients, %)

Complication	Lidocaine group (*n* = 30)	Placebo group (*n* = 30)	*p* value
Ileus, *n* (%)	3 (10.0 %)	5 (16.7 %)	0.706
Nausea, *n* (%)	5 (16.7 %)	6 (20.0 %)	1.000
Vomiting, *n* (%)	3 (10.0 %)	2 (6.7 %)	1.000
Urinary retention, *n* (%)	0	2 (6.7 %)	0.492
Anastomotic leak, *n* (%)	1 (3.3 %)	1 (3.3 %)	1.000
Wound infection, *n* (%)	0	1 (3.3 %)	1.000

## Discussion

Despite a slow start, there has recently been a substantial increase in the number of units performing laparoscopic colorectal surgery for benign and malignant disease [14, 15]. Laparoscopic colon resection was introduced in 1991 [16, 17]. Large comparative studies and multiple prospective randomized control trials have reported equivalence in resection margin, lymph node collection, tumour recurrence, post-operative complications, and long-term outcomes between open and laparoscopic resection for colon cancer [18, 19]. In addition, these studies demonstrated earlier recovery of bowel function, less post-operative pain, and decreased length of stay with the laparoscopic approach which has heralded widespread acceptance for laparoscopic resection of colon cancer [20, 21].

A standard method of post-operative analgesia for patients undergoing laparoscopic colorectal surgery has not yet been developed, and this has led to the employment of various types of analgesia. These have included epidural, intrathecal analgesia, patient-controlled analgesia, and IV agents.

More recently, Kaba et al. [7] reported the use of IV lidocaine for post-operative pain control. They found a significantly shorter length of hospital stay, less time to passing flatus and to the first bowel movement, and better pain scores in patients who received IV lidocaine. The doses that were used were equivalent to doses previously used for cardiac arrhythmias. These doses were administered on the ward, post-operatively, with no complications. Schlachta et al. [22] reported that use of IV ketorolac rather than placebo significantly reduced the length of hospital stay, as did the use of IV lidocaine compared to placebo.

The analgesic and anti-inflammatory effects of systemic lidocaine administration may be a result of block or inhibition of nerve conduction [13]. This is related to the abilities of systemic lidocaine to depress spike activity, amplitude, and conduction time in both myelinated A–C and unmyelinated C fibres significantly [23]. In addition, it has been shown that IV lidocaine decreased the heat-I capsaicin-induced secondary hyperalgesia via its central effect, which also suppressed secondary hyperalgesia in experimental incision-induced pain by inhibiting centralization [24, 25].

Lidocaine toxicity follows a predictable progression and can be divided into central nervous system and cardiovascular effects. At low plasma concentrations, these include numbness of the tongue and perioral tissues. If plasma concentrations reach higher levels, restlessness, vertigo, tinnitus, and accommodation disorder may be apparent. Other adverse reactions caused by high concentrations may involve slurred speech, skeletal muscle twitching, and drowsiness followed by seizures. Although the side effects are dose-dependent, they are more frequent at higher infusion rates (more than 3 mg/min). In this study, lidocaine was infused at 1 mg/min for 24 h, and there were no adverse reactions. Lidocaine toxicity tends to occur at high plasma concentrations (more than 5 mkg/mL) and has not been seen even when infused during longer periods [26].

In previous studies [7, 9, 10, 13, 27, 28], it has been suggested that lidocaine infusion (the doses and length of treatment differ) is safe and causes no serious side effects. However, perioral numbness and tinnitus were reported in one study with IV lidocaine pain management [29]. Therefore, medical observation may be required as long as the lidocaine infusion is continued.

The results of this study indicate that perioperative use of IV lidocaine significantly reduces pain after surgery compared with placebo in laparoscopic colorectal surgery.

Previous studies of visceral surgery [7, 9, 10, 13, 27, 28] examined post-operative outcomes with systemic lidocaine infusion from one to 24 h after intraoperative infusion. Perioperative lidocaine infusion provided better pain relief after radical prostatectomy [13], laparoscopic colectomy [7], and colon surgery [28], whereas in a study by Koppert et al. [9] and one by Herroeder et al. [10], lidocaine infusion until 1 h after major abdominal surgery and until 4 h after colorectal surgery, respectively, did not show a preventive effect on post-operative pain intensity. Nevertheless, lidocaine infusion of longer duration appears to provide the most significant impact on post-operative morbidity and length of hospital stay. In a study by Kaba et al. [7], the authors suggested that the prolonged infusion of lidocaine for 24 h following intraoperative infusion resulted in significantly improved outcomes for all study parameters such as pain scores, opioid consumption, subjective feeling of fatigue, return of bowel function, and length of hospital stay after laparoscopic colectomy.

Therefore, we suggest that the impact of perioperative low-dose lidocaine infusion on length of hospital stay was of a true benefit, consistent with previous results [7, 10, 13]. Earlier discharge of patients who received lidocaine was suggested to be related to the rapid resolution of post-operative ileus [7, 13]. Due to anti-inflammatory effects of lidocaine, early recovery from ileus after surgery may be attributed to the administration of lidocaine.

LG patients proved to have quicker recovery of bowel function as evidenced by earlier time of first flatus as well as first bowel movement. In our study, LG patients had a bowel movement more than 24 h earlier than PG patients and were discharged 1.2 days earlier.

The findings of Wongyingsinn et al. [30] and Swenson et al. [31] demonstrate that perioperative IV infusion of lidocaine has the same impact on post-operative restoration of bowel function as epidural analgesia, with an equal incidence of complications and duration of hospital stay. This suggests that systemic lidocaine administration may be an appropriate alternative to epidural therapy, particularly in settings in which epidural placement is technically difficult, contraindicated, or undesired. This less invasive approach to post-operative management would most likely be simpler and potentially safer than epidural administration of local anaesthetic.

Post-operative nausea and vomiting are substantial problems, since even mild nausea and vomiting can delay discharge from hospital, leading to increased costs and decreased patient satisfaction [31]. The present study showed a benefit of IV lidocaine infusion in terms of nausea, which might have been derived from of the anti-inflammatory and propulsive effects of lidocaine. There was, however, no statistically significant effect on nausea or vomiting.

It remains unclear why patients undergoing abdominal surgery may particularly benefit from IV lidocaine infusion. One explanation might be the differences in pain mechanisms; visceral pain from abdominal surgery might be triggered by mechanisms other than those involved in non-visceral pain. Part of the discomfort and pain following abdominal surgery might be related to post-operative ileus and nausea/vomiting. Indeed, the reduction observed in the time to bowel function recovery as well as the decreased incidence of nausea/vomiting might explain why the improvement in pain control was observed mainly in studies based on abdominal surgery.

There are some limitations associated with this study. The present study analyzed pain scores and ketorolac usage during the first 24 h after surgery at the same time patients were receiving continuous infusion of 0.1 μg/kg/h fentanyl. No data were collected on pain scores and analgesic usage on the further days until discharge. Ketorolac usage by patients in terms of pain scores and time was not analyzed. Although our study showed advantages of IV lidocaine in post-operative pain relief, we failed to show its superiority compared with epidural anaesthesia. Also, our study did not provide any data concerning cost-effectiveness. To clarify both items, further studies are needed. Finally, out prospective randomized trial was not registered with ClinicalTrials.gov.

## Conclusions

Continuous IV lidocaine infusion has a beneficial effect in patients who have undergone HALS and decreases post-operative pain, facilitating early restoration of bowel function and shortening length of hospital stay.
